# Supplementary material for: Comparing migration of Whinchats Saxicola rubetra from the non-breeding grounds in Liberia and Nigeria: Differences due to geography but otherwise very similar
Source: PLoS One. 2025 Jun 2;20(6):e0324086. doi: 10.1371/journal.pone.0324086 (PMC12129333; doi:10.1371/journal.pone.0324086)
Supplement: S1 Table — (DOCX) [file pone.0324086.s001.docx]

**S1 Table: Comparison of biometrics between Nigerian and Liberian whinchats used in analyses.**

|  | Intercept |  |  | Difference |  |  |  |  |
| --- | --- | --- | --- | --- | --- | --- | --- | --- |
|  | Liberia |  |  | Nigeria |  | t value | P value | dof |
| Mass g +/- SE | 15.9 | 1.0 |  | 0.2 | 1.1 | 0.1 | 0.89 | 58 |
| Wing length mm +/- SE | 77.2 | 0.4 |  | 0.9 | 0.5 | 1.9 | 0.054 | 62 |
| Pectoral score +/- SE | 1.6 | 0.2 |  | -0.3 | 0.2 | -1.2 | 0.20 | 62 |
| Tarsus length mm +/- SE | 22.4 | 0.3 |  | 0.2 | 0.3 | 0.5 | 0.64 | 60 |
