# Supplementary material for: Comparing migration of Whinchats Saxicola rubetra from the non-breeding grounds in Liberia and Nigeria: Differences due to geography but otherwise very similar
Source: PLoS One. 2025 Jun 2;20(6):e0324086. doi: 10.1371/journal.pone.0324086 (PMC12129333; doi:10.1371/journal.pone.0324086)
Supplement: S2 Table — (DOCX) [file pone.0324086.s002.docx]

**S2 Table: Raw data summary**

|  | Min | Mean | Max | N |
| --- | --- | --- | --- | --- |
| Spring migration distance km | 4164 | 5936 | 7733 | 51 |
| Autumn migration distance km | 4558 | 6025 | 7877 | 41 |
| Migration leg distance km | 262 | 2144 | 6038 | 278 |
| Migratory leg duration days | 1 | 3.1 | 24 | 278 |
| No. of stopovers days | 1 | 2.8 | 5 | 92 |
| Duration of stopovers days* | 2 | 8.8 | 54 | 186 |
| Overall spring migration duration days* | 7 | 21.6 | 40 | 51 |
| Overall autumn migration duration days | 16 | 36.9 | 113 | 40 |
| Departure date non-breeding* | 27-Mar | 17-Apr | 02-May | 64 |
| Arrival date at breeding site | 23-Apr | 08-May | 28-May | 51 |
| Duration on breeding site days | 38 | 108 | 145 | 42 |
| Departure date breeding site | 10-Jun | 24-Aug | 27-Sep | 42 |
| Arrival date non-breeding site | 04-Sep | 30-Sep | 30-Oct | 40 |
| Breeding latitude | 43.4 | 55.3 | 69.6 | 42 |
| Breeding longitude | 7.5 | 29.8 | 48.6 | 42 |
|  |  |  |  |  |
| * Excluding sub-Saharan movements or stationary periods prior to spring crossing of the Sahara | | | | |
