# Supplementary figures and images for: Comparing migration of Whinchats Saxicola rubetra from the non-breeding grounds in Liberia and Nigeria: Differences due to geography but otherwise very similar
Source: PLoS One. 2025 Jun 2;20(6):e0324086. doi: 10.1371/journal.pone.0324086 (PMC12129333; doi:10.1371/journal.pone.0324086)

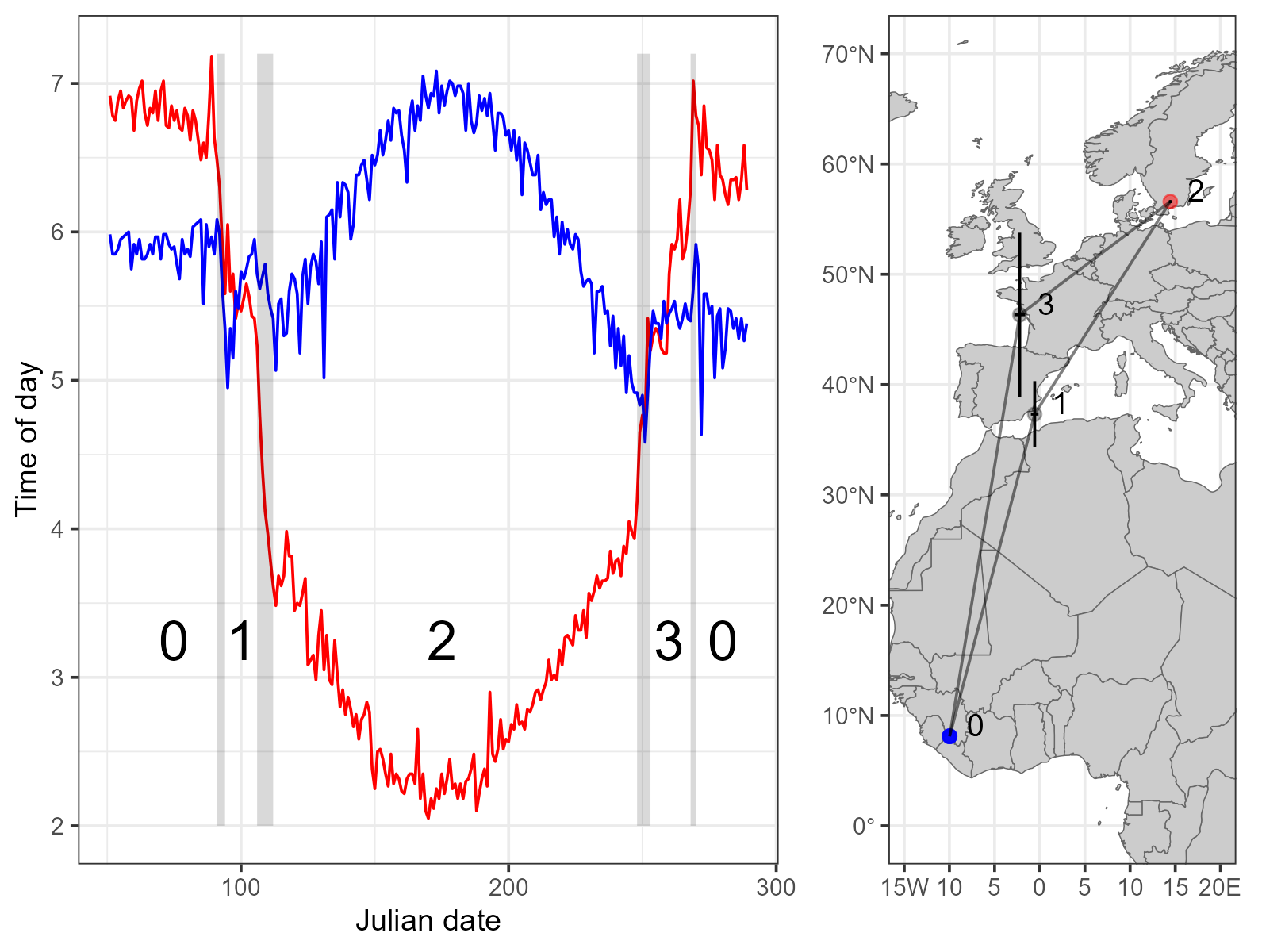

Supplement: S1 Fig — (TIF) [file pone.0324086.s003.tif]
