# Supplementary material for: Comparing migration of Whinchats Saxicola rubetra from the non-breeding grounds in Liberia and Nigeria: Differences due to geography but otherwise very similar
Source: PLoS One. 2025 Jun 2;20(6):e0324086. doi: 10.1371/journal.pone.0324086 (PMC12129333; doi:10.1371/journal.pone.0324086)
Supplement: Attachment — Submitted filename: Response to Reviewers.docx [file pone.0324086.s005.docx]

PONE-D-25-07789

Comparing migration of Whinchats Saxicola rubetra from Liberia and Nigeria: behaviours modified by differences in geography but unlikely to be constrained by them

PLOS ONE

*Our responses below:*

Both reviewers agreed with the value of the dataset and were impressed with the sample size. They were, however, both unsure of some part of the methodology, in particular about handling of the geolocator data around the time of equinox and how you dealt with the uncertainty. Please clarify this, as well as responding to other suggestions made by the reviewers.

*Thank you for your prompt and helpful review. I have responded to all the referees’ comments fully and in detail as they suggested (in italics below), apart from a couple of places where the comments are subjective and less important. These are marked with NO CHANGE along with my argument to keep the text the same. None are deal breakers, simply differences in style of writing and I believe my approach is clearer in these cases.*

Please include the following items when submitting your revised manuscript:

- A rebuttal letter that responds to each point raised by the academic editor and reviewer(s). You should upload this letter as a separate file labeled 'Response to Reviewers'.

*Done*

- A marked-up copy of your manuscript that highlights changes made to the original version. You should upload this as a separate file labeled 'Revised Manuscript with Track Changes'.

*Done*

- An unmarked version of your revised paper without tracked changes. You should upload this as a separate file labeled 'Manuscript'.

*Done*

If you would like to make changes to your financial disclosure, please include your updated statement in your cover letter. Guidelines for resubmitting your figure files are available below the reviewer comments at the end of this letter.

*NA*

If applicable, we recommend that you deposit your laboratory protocols in protocols.io to enhance the reproducibility of your results. Protocols.io assigns your protocol its own identifier (DOI) so that it can be cited independently in the future. For instructions see: [https://journals.plos.org/plosone/s/submission-guidelines#loc-laboratory-protocols](https://eur01.safelinks.protection.outlook.com/?url=https%3A%2F%2Ftrack.editorialmanager.com%2FCL0%2Fhttps%3A%252F%252Fjournals.plos.org%252Fplosone%252Fs%252Fsubmission-guidelines%2523loc-laboratory-protocols%2F1%2F010f0195acedd0db-f5c38916-7c3b-4462-a52f-a04f029154d4-000000%2FqXVIhsbLOncoaHSyX81IIW5RUj7FvOz6rzJpZCqaP5c%3D203&data=05%7C02%7Cwrlc%40st-andrews.ac.uk%7Ce53245a9a1644760d50008dd66a959ca%7Cf85626cb0da849d3aa5864ef678ef01a%7C0%7C0%7C638779600024141865%7CUnknown%7CTWFpbGZsb3d8eyJFbXB0eU1hcGkiOnRydWUsIlYiOiIwLjAuMDAwMCIsIlAiOiJXaW4zMiIsIkFOIjoiTWFpbCIsIldUIjoyfQ%3D%3D%7C0%7C%7C%7C&sdata=UXuMN%2BWIfUKR0fdtOJ4te%2F4rHdYZ%2BQgUQsyawqcyGKc%3D&reserved=0). Additionally, PLOS ONE offers an option for publishing peer-reviewed Lab Protocol articles, which describe protocols hosted on protocols.io. Read more information on sharing protocols at [https://plos.org/protocols?utm_medium=editorial-email&utm_source=authorletters&utm_campaign=protocols](https://eur01.safelinks.protection.outlook.com/?url=https%3A%2F%2Ftrack.editorialmanager.com%2FCL0%2Fhttps%3A%252F%252Fplos.org%252Fprotocols%253Futm_medium%3Deditorial-email%2526utm_source%3Dauthorletters%2526utm_campaign%3Dprotocols%2F1%2F010f0195acedd0db-f5c38916-7c3b-4462-a52f-a04f029154d4-000000%2Fmbwomw_fYZ63cJG_OR3TmPdUEzT2bEKx_8tRI7uXBDQ%3D203&data=05%7C02%7Cwrlc%40st-andrews.ac.uk%7Ce53245a9a1644760d50008dd66a959ca%7Cf85626cb0da849d3aa5864ef678ef01a%7C0%7C0%7C638779600024166808%7CUnknown%7CTWFpbGZsb3d8eyJFbXB0eU1hcGkiOnRydWUsIlYiOiIwLjAuMDAwMCIsIlAiOiJXaW4zMiIsIkFOIjoiTWFpbCIsIldUIjoyfQ%3D%3D%7C0%7C%7C%7C&sdata=Rn05M7hhI4SASQhrBq%2F0Vyu1DOf2FCp%2FY6913pzNp2k%3D&reserved=0).

*NA*

Journal Requirements:

When submitting your revision, we need you to address these additional requirements.

1. Please ensure that your manuscript meets PLOS ONE's style requirements, including those for file naming. The PLOS ONE style templates can be found at

<https://journals.plos.org/plosone/s/file?id=wjVg/PLOSOne_formatting_sample_main_body.pdf>

*Done*

[https://journals.plos.org/plosone/s/file?id=ba62/PLOSOne_formatting_sample_title_authors_affiliations.pdf](https://eur01.safelinks.protection.outlook.com/?url=https%3A%2F%2Ftrack.editorialmanager.com%2FCL0%2Fhttps%3A%252F%252Fjournals.plos.org%252Fplosone%252Fs%252Ffile%253Fid%3Dba62%252FPLOSOne_formatting_sample_title_authors_affiliations.pdf%2F1%2F010f0195acedd0db-f5c38916-7c3b-4462-a52f-a04f029154d4-000000%2FQQzH7Z37DRoN0a3ewCrXzF8v0RLUaCbaWZXjhOSGCMY%3D203&data=05%7C02%7Cwrlc%40st-andrews.ac.uk%7Ce53245a9a1644760d50008dd66a959ca%7Cf85626cb0da849d3aa5864ef678ef01a%7C0%7C0%7C638779600024181391%7CUnknown%7CTWFpbGZsb3d8eyJFbXB0eU1hcGkiOnRydWUsIlYiOiIwLjAuMDAwMCIsIlAiOiJXaW4zMiIsIkFOIjoiTWFpbCIsIldUIjoyfQ%3D%3D%7C0%7C%7C%7C&sdata=YkxWyOSIXVXk9zQxrM16ZgEk7U7vYtUA8GSTaGePCsk%3D&reserved=0)

*Done although it was unclear how to allocate unequal authorship. Will Cresswell (me), designed and supervised the research, collected some of the data, analysed all the data, wrote the paper entirely; the rest of the team contributed to the bulk of the data collection and edited the paper.*

2**.** Thank you for stating the following financial disclosure:
“Chris Goodwin, A.P. Leventis Conservation Foundation, AP Leventis Ornithological Research Institute, the British Ornithologists’ Union and the Linnean Society”

Please state what role the funders took in the study. If the funders had no role, please state: "The funders had no role in study design, data collection and analysis, decision to publish, or preparation of the manuscript."

*Please regard the below as our statement:*

*“The funders had no role in study design, data collection and analysis, decision to publish, or preparation of the manuscript.”*

3. Thank you for stating the following in the Acknowledgments Section of your manuscript:
“This work was supported by Chris Goodwin, A.P. Leventis Conservation Foundation, AP Leventis Ornithological Research Institute, the British Ornithologists’ Union and the Linnean Society. This is paper number (to be completed at proof stage) from the AP Leventis Ornithological Research Institute”

Please remove any funding-related text from the manuscript

*Done*

and let us know how you would like to update your Funding Statement. Currently, your Funding Statement reads as follows:

“Chris Goodwin, A.P. Leventis Conservation Foundation, AP Leventis Ornithological Research Institute, the British Ornithologists’ Union and the Linnean Society”

*Please regard the below as our statement of funding:*

*“Chris Goodwin, A.P. Leventis Conservation Foundation, AP Leventis Ornithological Research Institute, the British Ornithologists’ Union and the Linnean Society”*

4. When completing the data availability statement of the submission form, you indicated that you will make your data available on acceptance. We strongly recommend all authors decide on a data sharing plan before acceptance, as the process can be lengthy and hold up publication timelines. Please note that, though access restrictions are acceptable now, your entire data will need to be made freely accessible if your manuscript is accepted for publication. This policy applies to all data except where public deposition would breach compliance with the protocol approved by your research ethics board. If you are unable to adhere to our open data policy, please kindly revise your statement to explain your reasoning and we will seek the editor's input on an exemption. Please be assured that, once you have provided your new statement, the assessment of your exemption will not hold up the peer review process.

*Done*

*The data and R script files has been deposited in the University of St Andrews open access data depository PURE with a DOI of:*

*Pure ID: 316400564 (The St Andrews Data Depository)*

*Title: Comparing migration of Whinchats Saxicola rubetra from the non-breeding grounds in Liberia and Nigeria: differences due to geography but otherwise very similar (dataset)*

*Inactive DOI:* [***https://doi.org/10.17630/59c8ff25-858d-4dcb-8f47-6b65894bd0e0***](https://eur01.safelinks.protection.outlook.com/?url=https%3A%2F%2Fdoi.org%2F10.17630%2F59c8ff25-858d-4dcb-8f47-6b65894bd0e0&data=05%7C02%7Cwrlc%40st-andrews.ac.uk%7Cc6cc22f4a1a44ca9342908dd6d1797cd%7Cf85626cb0da849d3aa5864ef678ef01a%7C0%7C0%7C638786670270601974%7CUnknown%7CTWFpbGZsb3d8eyJFbXB0eU1hcGkiOnRydWUsIlYiOiIwLjAuMDAwMCIsIlAiOiJXaW4zMiIsIkFOIjoiTWFpbCIsIldUIjoyfQ%3D%3D%7C0%7C%7C%7C&sdata=1ARgYykjrW1TGO4otMjroy6C0OAAUfi8If%2FZ4X5SzcE%3D&reserved=0)

*Once the manuscript is accepted and I have notified the University Research Depository office that the data files, in their final version, have been uploaded in Pure, they will proceed with the activation of the DOI.*

*Activation of the DOI will make the metadata for your dataset discoverable on the research portal. However, they will release the data files only once the article has been published online.*

5. Your ethics statement should only appear in the Methods section of your manuscript. If your ethics statement is written in any section besides the Methods, please move it to the Methods section and delete it from any other section. Please ensure that your ethics statement is included in your manuscript, as the ethics statement entered into the online submission form will not be published alongside your manuscript.

*Our ethics statement now appears at the end of the methods and nowhere else.*

6. We notice that your supplementary [figures/tables] are included in the manuscript file. Please remove them and upload them with the file type 'Supporting Information'. Please ensure that each Supporting Information file has a legend listed in the manuscript after the references list.

*All supporting information is now removed from the main text apart from the legend list at the end of the manuscript.*

*A file containing Supporting Information only has been made and uploaded*

[Note: HTML markup is below. Please do not edit.]

Reviewers' comments:

Reviewer's Responses to Questions

**Comments to the Author**

1. Is the manuscript technically sound, and do the data support the conclusions?

The manuscript must describe a technically sound piece of scientific research with data that supports the conclusions. Experiments must have been conducted rigorously, with appropriate controls, replication, and sample sizes. The conclusions must be drawn appropriately based on the data presented.

Reviewer #1: Yes

Reviewer #2: Partly

2. Has the statistical analysis been performed appropriately and rigorously?

Reviewer #1: Yes

Reviewer #2: Yes

3. Have the authors made all data underlying the findings in their manuscript fully available?

The [PLOS Data policy](https://eur01.safelinks.protection.outlook.com/?url=http%3A%2F%2Ftrack.editorialmanager.com%2FCL0%2Fhttp%3A%252F%252Fwww.plosone.org%252Fstatic%252Fpolicies.action%2523sharing%2F1%2F010f0195acedd0db-f5c38916-7c3b-4462-a52f-a04f029154d4-000000%2FWcysyn-Ep9xArQzenCJirNAryqtWadqgCycz91cH4C8%3D203&data=05%7C02%7Cwrlc%40st-andrews.ac.uk%7Ce53245a9a1644760d50008dd66a959ca%7Cf85626cb0da849d3aa5864ef678ef01a%7C0%7C0%7C638779600024195876%7CUnknown%7CTWFpbGZsb3d8eyJFbXB0eU1hcGkiOnRydWUsIlYiOiIwLjAuMDAwMCIsIlAiOiJXaW4zMiIsIkFOIjoiTWFpbCIsIldUIjoyfQ%3D%3D%7C0%7C%7C%7C&sdata=myjmCQR8oVBPBw9PR2Xrved1oWVbWbj7jtx3JgRd%2FEs%3D&reserved=0) requires authors to make all data underlying the findings described in their manuscript fully available without restriction, with rare exception (please refer to the Data Availability Statement in the manuscript PDF file). The data should be provided as part of the manuscript or its supporting information, or deposited to a public repository. For example, in addition to summary statistics, the data points behind means, medians and variance measures should be available. If there are restrictions on publicly sharing data—e.g. participant privacy or use of data from a third party—those must be specified.

Reviewer #1: No

Reviewer #2: No

*DONE as above*

*The data and R script files has been deposited in the University of St Andrews open access data depository PURE with a DOI*

4. Is the manuscript presented in an intelligible fashion and written in standard English?

PLOS ONE does not copyedit accepted manuscripts, so the language in submitted articles must be clear, correct, and unambiguous. Any typographical or grammatical errors should be corrected at revision, so please note any specific errors here.

Reviewer #1: Yes

Reviewer #2: Yes

5. Review Comments to the Author

Please use the space provided to explain your answers to the questions above. You may also include additional comments for the author, including concerns about dual publication, research ethics, or publication ethics. (Please upload your review as an attachment if it exceeds 20,000 characters)

Reviewer #1: Review – Plos One

Comparing migration of Whinchats Saxicola rubetra from Liberia and Nigeria: behaviours modified by differences in geography but unlikely to be constrained by them

In this article, the authors describe the migration strategies of Whinchat, tagging birds from two non-breeding sites, allowing an unprecedented comparison with an impressive sample size for such a small migrant. This article provides valuable insight into the migration of this species, but also very important data that can apply to the Afro-palearctic migratory system in general, provided that more research will be carried out using a similar design. This work is worth publishing in Plos One and will be of interest for the specialized readership as well as for readers interested in more general novelty in biological research.

I listed minor suggestions hereafter. The only major remark concerns the visual classification method (see under methods), and hereby the limitations of the light-level geolocation to classify migration legs. This should be better explained in the methods, including more precisions on the general uncertainties, and some caution should be taken while presenting migration leg speed for example.

*Corrections made as per Referee’s suggestions and are detailed below under the specific points*

Title: I would suggest rephrasing the title in a shorter, simpler and clearer way (the second part takes time to associate to its meaning, at least for me). Mention could be made in the title that the study is based on non-breeding sites, because the “from” could be misleading for the non-specialized readership and because this is what makes this study more interesting.

*Changed as suggested to:*

*Comparing migration of Whinchats Saxicola rubetra from the non-breeding grounds in Liberia and Nigeria: differences due to geography but otherwise very similar*

Abstract
L. 19: “whinchats” plural?

*Changed as suggested.*

L. 19: rephrasing suggestion: “a declining palearctic-breeding passerine” for example.

*Changed as suggested*

L. 21: not clear what is meant with differences in migratory connectivity between the two sites (connectivity is the fact that two non-breeding populations would go to two distinct breeding areas?) maybe reframe and put the connectivity argument into one separate sentence?

*Changed as suggested to:*

*We predicted differences, resulting from the geographical location of the two non-breeding sites, in location of respective breeding areas (migratory connectivity),*

L. 23: “distance, duration…” (not necessary to mention speed, as it is here derived from distance and duration, no data on real speed in the air is shown).

*Deleted as suggested*

L. 24 not clear from the sentence in the abstract why these two populations should have a different number of stopover sites (and not sure this is important for the abstract).

*NOT CHANGED*

*I disagree. Number of stop-overs is probably what really determines survival in long distance migrants as each reflects a site that must be suitable for the migration to succeed (the chain-link hypothesis). That Liberian populations have longer migrations and so more stop overs might mean that these populations are declining while Nigeria populations aren’t – so this is important.*

L. 28: “significant loop migration” : to avoid confusion, I suggest omitting this unnecessary “significant” unless related so statistical analyses here

*Clarified as suggested.*

*“Liberian birds had longer migrations in distance and duration, and more stopovers, but only Nigerian birds showed a statistically significant difference in longitude comparing spring and autumn migrations (i.e. a clear loop migration).”*

L. 35: “able to adapt to potential climate change across Europe and West Africa”: this conclusion is not well supported by the data; I would be more cautious with mentioning this like that here.

*Modified to be clearly speculative – but it is an important and I think probably correct point, even if our results are only concordant with this conclusion.*

*“This suggests whinchats are well adapted to the current variable geography and* ***so may have the capacity*** *to adapt to potential climate change across Europe and West Africa, although average quality and availability of stopover sites may be contributing to declines.”*

Introduction
L. 41: probably true. However, most of these birds eat insects and this should be mentioned as a potential cause of decline, if the topic of the cause of decline is approached (not necessarily the core topic of this paper). Furthermore, declining long-distance migrants that eat seeds are persecuted (ortolan, turtle doves) and also decline. So there is a broad spectrum of reasons why these birds decline (with some exceptions locally), but discussing this here might not be needed.

*NO CHANGE MADE*

*I don’t think we need to go into the detail here, especially in the very general first paragraph, and anyway, broadly speaking, the referee seems ok with the original text.*

L. 46: “to such changes in the non-breeding area “?

*Added as suggested*

L. 49: I disagree with this expectation as it is phrased (many species have different migration strategies across populations, see blackcap for example), but this might be related to the phrasing.

*Rephrased as suggested:*

*“****Some*** *migration characteristics should be similar across all populations of a species”*

Methods:
L. 205-209: I have some doubts about the accuracy of visual inspection method, as whinchats migrate on a mostly north-south axis and at times close to equinox (especially for the post-breeding migration). Could you provide some more details and quantify uncertainties, even broadly? Are there situations where migration periods could not be assessed accurately? I would be surprised if not.

*Additional detail added as suggested; in short sensitivity analysis indicates that the few equinoxial movements make no difference to the results.*

*Added text, one paragraph up from data analysis subheading, methods section:*

*“Geolocator locations are also subject to imprecision calculating latitude at the equinox periods (the few days either side of March 20^th^ and September 22^nd^ when regardless of latitude, daylength is similar). Although latitude is unreliable during these periods, estimation of longitude, that depends on sunrise and sunset times, remains consistently accurate. Assessment of migration phenology also remains consistently accurate, except in the very rare cases where a migration leg both occurs close to the equinox and broadly follows a north-south axis, without any longitudinal change so that both sunrise and sunset times would not be seen to change on any visual examination of the data. There were 16 migration periods that occurred within one week of the equinox and the mean change in longitude was 9.3 degrees, with a minimum value of 4.2 degrees. In terms of change of sunrise and sunset times, both would shift by at least approximately 17 minutes for the minimum change in longitude we observed, large enough to be detected by graphical inspection (see Appendix Figure 1), and so allowing the migration period to be identified, if not the accurate latitude of the stationary periods either side of it. In any case most of the migration periods within a week of the equinox (10/16) involved sunrise or sunset shifts of more than one hour. Furthermore, only a few of the hypotheses tested in this paper are affected by this imprecision in calculating latitude caused by migration close to equinox (see Prediction 5 below), and in these cases analyses were also run excluding the data from the equinox periods to determine if there was any effect of the increased uncertainty in the data during these periods.”*

*Added to the methods for Prediction 2:*

*“None of the migration periods to change non-breeding sites south of the Sahara occurred within one week of the equinox.”*

*Added to the methods for Prediction 3:*

*“This analysis was unaffected by any imprecision in latitude that might arise at the equinox because any over or underestimates in latitude for a stopover period, would cancel out and so broadly not affect the overall total migration distance between the main non-breeding site in Africa and the breeding site in Europe.”*

*Added to the methods for Prediction 5:*

*“Analyses were also run excluding the 16 migration and stopover periods that occurred within one week of the equinox to determine how imprecision in calculation of latitude during these periods affected the results.”*

*Added to the results for Prediction 5:*

*“There was no significant variation in the distance migrated in a leg by country or season, or their interaction: on average whinchats flew 2,043 ± 67 km (N = 298 distances from 64 individuals) per migration leg* ***(this was 2,053 ± 69 km excluding the 16 equinox samples)****.*

*And*

*“There was no significant variation in the duration of a migration leg by country or season, or their interaction: a migration leg lasted on average 3.1 ± 0.1 days (N = 298 durations from 64 birds; the null model was best supported: ΔAIC = -3.1 compared to the main effects model including country and season).* ***Excluding the 16 equinox samples (3.1 ± 0.1 days)*** *or excluding sub-Saharan stopovers prior to spring migration gave very similar results (Table 1).”*

L. 289: it is likely that there were stopovers of less than two days, but they were not detected with the classification method. Stopovers of one day are very common in other species while crossing the desert and the Mediterranean (see for example <https://doi.org/10.1186/s40462-023-00381-6>, which refers to a closely related species that performs such stops of less than a day, for example). I expect such short stops to occur in Whinchats at least partly: could you give more details about this?

*Further details added as suggested to 2^nd^ paragraph, data processing:*

*“Note that stopover periods of less than two days, including daily stopovers of a few hours that may occur regularly during migration legs cannot be discerned from geolocator data except by a lower speed of travel within a leg than expected if a passerine was travelling continuously at expected average flight speed (see Discussion section 5).”*

L.298-299: I might have missed it, but did you set a time threshold to consider a non-breeding site? There are often stop over sites located close to non-breeding sites, but with only a few days I would not consider them as alternative non-breeding sites but rather stopover sites within the non-breeding region.

*Further details of time threshold added as suggested to methods, Prediction 2:*

*“We plotted all stationary periods south of the Sahara and away from the tagging sites (all were over 250 km away), of more than 2 days (range 13 – 52 days, apart from one of 3 days, representing a third non-breeding site, that was included because it followed a second non-breeding site that was occupied for 19 days, and both involved substantial movements in the opposite direction to subsequent migration to the breeding ground), as an additional non-breeding site (see Figure 3).”*

Results:

L. 353: did you have locations on the sea, and did you use a sea mask to correct for this?

*Some locations are on the sea, although none are on the sea without some of their confidence intervals overlapping land. A sea mask was not used because they do not account for error evenly across a migration; they bias the results in terms of accuracy for migrations that fly over more sea and they do not take into account the very, very many small islands that occur in the Mediterranean. In short I have no confidence in sea masks and don’t use them. I don’t want to get into the argument about sea masks in this paper – it needs a proper methodological focussed paper. In short – the whole analysis approach in this paper is to assume errors are unbiased and then approach the truth through averages. Sea masks would change this approach.*

L. 354: is the spatial precision sufficient to qualify the country in which the bird was, or should you stay with more general areas/directions?

“The subsequent flight for this bird crossed from the southern coast of Ghana to the
Mediterranean coast of Tunisia in a single leg of 3,538 km over a 5-day period”
If referring to more precise migration data in other species (<https://doi.org/10.1186/s40462-023-00381-6> for example), it is likely that this bird landed over the day at least some hours and did not fly non-stop (in which case it would likely have taken max. 3 days), this could be explained better and discussed in the discussion section. It would be awesome to fit pressure loggers on Whinchats in the future to refine this (could be mentioned in the discussion).

*Agreed, and more speculative phrasing added as suggested; also rewritten with respect to comment below.*

*“The overall total maximum distance of movement recorded was* ***approximately*** *1,442 km (note the latitudinal part of this will be subject to location error) by a bird that moved south* ***approximately*** *to the coast of Nigeria and then* ***approximately*** *to north-west to Ghana, spending 35 days and 17 days at the two sites respectively; a second bird also* ***approximately*** *reached Ghana, travelling southwest, 356 km and 765 km in two legs, with a stopover of 2 and 19 days respectively. The subsequent flight for this bird crossed from* ***approximately*** *the southern coast of Ghana to* ***approximately*** *the Mediterranean coast of Tunisia in a single leg of around 3,538 km over a 5-day period. The total distance travelled between the main Nigerian non-breeding site and the Mediterranean coast for this bird was therefore* ***approximately*** *4,659 km,* ***approximately*** *2,130 km further than the direct, great circle distance from its main non-breeding site in Nigeria to where it eventually arrived on the Mediterranean coast. One final bird also used multiple sites. The bird was tagged in 2013 was only recaptured in 2014, 18 months after tagging. This bird spent the entire non-breeding period of 2013-14, at least until March 25^th^ 2014 when the logger battery ran out, at a site about 720 km to the west* ***approximately*** *on the border of Chad and Cameroon.”*

L. 414: for discussion: could this be related to the onset of rains in Nigeria in the northern spring, from which Whinchats could benefit to refuel before crossing the desert (while rainfalls are less strongly seasonal in Liberia and start earlier?)

*NO CHANGE*

*I wouldn’t want to get into this because we only have one year for Liberia and onset of rains are highly variable between years even for Liberia.*

L. 447-453: I suggest skipping or being more cautious about the estimate of migration speed, as it is likely biased by the classification method and not a very important finding of the study.

*A more cautious approach taken throughout as suggested, and as per comment below.*

L. 625: this partly solves my previous remark, but I would make it clearer in methods and results that the speed you measure is including some stationary phases. Since this migration leg speed is not a key finding of the study, I would give it less importance overall in the paper.

*Changed as suggested.*

*I have now made clearer in the methods (see also comment above)*

*Further details added as suggested to 2^nd^ paragraph, data processing:*

*“Note that stopover periods of less than two days, including daily stopovers of a few hours that may occur regularly during migration legs cannot be discerned from geolocator data except by a lower speed of travel within a leg than expected if a passerine was travelling continuously at expected average flight speed (see Discussion section 5).”*

*And to Results Section 5:*

*Overall, an average speed of travel* ***(note this is not flying speed because it also includes stationary periods of less than 2 days)*** *during a migration leg of 2043 km/ (3.1 days x 24 hours) = 27.5 km/h was therefore reasonably representative across populations and seasons.*

L. 685: I find this example of extremely short total migration time very interesting, could it be better highlighted in the results and maybe even in the abstract?

*Agreed. Added to abstract and results as suggested:*

*One whinchat moved from sub-Saharan non-breeding site to northern European breeding site in 7 days, and 8% of complete migration durations were 14 days or less.*

L. 694 – 695: probably not true for some desert stops?

*Modification added as suggested*

*“The whinchat must have encountered good foraging conditions at each daily minor stopover site that allowed sufficient refuelling, including potentially, oases during its crossing of the Sahara.”*

Figures
On figure 3 especially, but also other figures, a visual representation of uncertainties could be valuable, especially for supposedly southwards movements before migration.

*One standard error added to location plots for Figure 2 and Figure 3 as suggested, but not Figure 1 because the figure is already hard to see detail on and heading towards cluttered.*

Thanks for this very interesting read.

*Thank you for the helpful comments*

Reviewer #2: In this study, authors clarified the characteristics of migration strategies of the whinchat, comparing them with two non-breeding populations. Authors focused on the 5 main topics of the two populations, i.e., 1) breeding ranges, 2) secondary non-breeding sites, 3) migration distance, the number of stopover sites and loop migration, 4) migration duration and phenology and 5) migration leg and stopover duration. The large dataset of whinchat’s migration presented some convincing results, but some problems should be corrected. My comments are listed below.

L130-131　
I think that there is insufficient evidence to provide a hypothesis that loop migration does not occur in Liberian wintering population. Please provide additional information regarding the reasoning behind your hypothesis.

*Agreed, there could always be a loop migration but geography (lack of land to the west for Liberian birds) is likely to limit this. I have rewritten this section to be more speculative, and to say we test for a difference because there is less room for a loop migration starting from Liberia.*

*“Liberian birds will have longer total migration distances, more stopovers and much less of a loop migration, if at all.”*

L193
How did you treat the geolocator data during the equinox period? Tracked individuals appear to depart or arrive during the equinox period, as shown in Table S1. However, there is no description of the data analysis for this period.

*Additional detail added as suggested; in short sensitivity analysis indicates that the few equinoxal movements make no difference to the results.*

*Added text, one paragraph up from data analysis subheading, methods section:*

*“Geolocator locations are also subject to imprecision calculating latitude at the equinox periods (the few days either side of March 20^th^ and September 22^nd^ when regardless of latitude, daylength is similar). Although latitude is unreliable during these periods, estimation of longitude, that depends on sunrise and sunset times, remains consistently accurate. Assessment of migration phenology also remains consistently accurate, except in the very rare cases where a migration leg both occurs close to the equinox and broadly follows a north-south axis, without any longitudinal change so that both sunrise and sunset times would not be seen to change on any visual examination of the data. There were 16 migration periods that occurred within one week of the equinox and the mean change in longitude was 9.3 degrees, with a minimum value of 4.2 degrees. In terms of change of sunrise and sunset times, both would shift by at least approximately 17 minutes for the minimum change in longitude we observed, large enough to be detected by graphical inspection (see Appendix Figure 1), and so allowing the migration period to be identified, if not the accurate latitude of the stationary periods either side of it. In any case most of the migration periods within a week of the equinox (10/16) involved sunrise or sunset shifts of more than one hour. Furthermore, only a few of the hypotheses tested in this paper are affected by this imprecision in calculating latitude caused by migration close to equinox (see Prediction 5 below), and in these cases analyses were also run excluding the data from the equinox periods to determine if there was any effect of the increased uncertainty in the data during these periods.”*

*Added to the methods for Prediction 2:*

*“None of the migration periods to change non-breeding sites south of the Sahara occurred within one week of the equinox.”*

*Added to the methods for Prediction 3:*

*“This analysis was unaffected by any imprecision in latitude that might arise at the equinox because any over or underestimates in latitude for a stopover period, would cancel out and so broadly not affect the overall total migration distance between the main non-breeding site in Africa and the breeding site in Europe.”*

*Added to the methods for Prediction 5:*

*“Analyses were also run excluding the 16 migration and stopover periods that occurred within one week of the equinox to determine how imprecision in calculation of latitude during these periods affected the results.”*

*Added to the results for Prediction 5:*

*“There was no significant variation in the distance migrated in a leg by country or season, or their interaction: on average whinchats flew 2,043 ± 67 km (N = 298 distances from 64 individuals) per migration leg* ***(this was 2,053 ± 69 km excluding the 16 equinox samples)****.*

*And*

*“There was no significant variation in the duration of a migration leg by country or season, or their interaction: a migration leg lasted on average 3.1 ± 0.1 days (N = 298 durations from 64 birds; the null model was best supported: ΔAIC = -3.1 compared to the main effects model including country and season).* ***Excluding the 16 equinox samples (3.1 ± 0.1 days)*** *or excluding sub-Saharan stopovers prior to spring migration gave very similar results (Table 1).”*

L252-267
This paragraph should be moved to the result section.

*NOT CHANGED.*

*I disagree. Even if the rules of writing paper state that sample size information is best put in the results section, I invoke George Orwell’s 12 rules of writing, in which Rule Number 12 states “if you can better and more elegantly communicate then break any of the rules 1 – 11”. The information informs the rest of the data analysis section so needs to come where I put it.*

L276
‘therefore do not include a random effect.’
Here, please add a sentence such as ‘i.e., analysed using Generalized Linear Models’.

*Added as suggested*

L277, L433-441
Model selection and statistical tests should not be conducted simultaneously because their objectives are different. I think that model selection based on AIC/AICc is unnecessary because the focus, as in the other analyses, was on the significance of the explanatory variable (including the interaction term).

*NOT CHANGED*

*Both the Information Theoretic approach and the Probabilistic approach have their advantages and disadvantages, and these change dependent on whether you are aiming to identify the simplest model or testing a priori hypotheses. There are both aims in this paper and so both approaches. This philosophical point of view – one approach or the other, never both in a paper – is one of opinion and fashion rather than based on anything practical. To use an analogy – why ignore an eye witness statement at a crime scene, even if you have a lot of compelling forensic data?*

L377
‘-.0’: is this correct?

*Typo now corrected to say:*

*“-1.0”*

L358-360
I could not understand how the distance was calculated. Please explain it more precisely.

*I have rewritten this completely for clarity:*

*“The subsequent flight for this bird crossed from approximately the southern coast of Ghana to approximately the Mediterranean coast of Tunisia in a single leg of around 3,538 km over a 5-day period. The total distance travelled between the main Nigerian non-breeding site and the Mediterranean coast for this bird was therefore approximately 4,659 km, approximately 2,130 km further than the direct, great circle distance from its main non-breeding site in Nigeria to where it eventually arrived on the Mediterranean coast.”*

L440
I think that P=0.11 is not marginal.

*Apologies, marginal deleted as suggested.*

L568　
Figure 2? I think Figure 1 is correct here.

*Typo, corrected to Figure 1 as suggested, but now deleted -see below*

L568
‘Our Liberia results possibly show this trend as well’ I think that it is over discussion here.

*Deleted as suggested.*

L633-634
Replace [ ] with ( ).

*Changed as suggested*

L1010
I think that the line colors of the minimum convex polygons are reversed between the Liberian and Nigerian populations in Figure 2.

*Detail added to clarify.*

*Yes, this is deliberate to improve contrast where they overlap; the different areas are now flagged in the legend to remove any doubt about the colour codes.*

L1034　
It seems that the table is misaligned. Is it correct?

*Alignment corrected as suggested*

While revising your submission, please upload your figure files to the Preflight Analysis and Conversion Engine (PACE) digital diagnostic tool, [https://pacev2.apexcovantage.com/](https://eur01.safelinks.protection.outlook.com/?url=https%3A%2F%2Ftrack.editorialmanager.com%2FCL0%2Fhttps%3A%252F%252Fpacev2.apexcovantage.com%252F%2F1%2F010f0195acedd0db-f5c38916-7c3b-4462-a52f-a04f029154d4-000000%2F_pgGonHtmkUN-95poIYqVxiBLypVkwK3XETxsTY-BRo%3D203&data=05%7C02%7Cwrlc%40st-andrews.ac.uk%7Ce53245a9a1644760d50008dd66a959ca%7Cf85626cb0da849d3aa5864ef678ef01a%7C0%7C0%7C638779600024239209%7CUnknown%7CTWFpbGZsb3d8eyJFbXB0eU1hcGkiOnRydWUsIlYiOiIwLjAuMDAwMCIsIlAiOiJXaW4zMiIsIkFOIjoiTWFpbCIsIldUIjoyfQ%3D%3D%7C0%7C%7C%7C&sdata=SmvFmEellBgnfzUpiHWd1kpjj5HBM1dhgROnlFZVq44%3D&reserved=0). PACE helps ensure that figures meet PLOS requirements. To use PACE, you must first register as a user. Registration is free. Then, login and navigate to the UPLOAD tab, where you will find detailed instructions on how to use the tool. If you encounter any issues or have any questions when using PACE, please email PLOS at [figures@plos.org](mailto:figures@plos.org). Please note that Supporting Information files do not need this step.

*Done. All figures processed and corrected at PACE and then downloaded; I have submitted the PACE processed figures*

*I believe we have addressed all of the significant points completely in the spirit that the Editor and Referees intend, and have formatted the manuscript, figures, and supporting information as instructed, and made the appropriate preparations for public deposit of our research data when the paper is finally accepted.*

*Thank you for all of your hard work on the manuscript.*

*Will Cresswell 27^th^ March 2025*
